# Associations between detectable circulating tumor DNA and tumor glucose uptake measured by ^18^F-FDG PET/CT in early-stage non-small cell lung cancer

**DOI:** 10.1186/s12885-023-11147-z

**Published:** 2023-07-11

**Authors:** Anine Larsen Ottestad, Håkon Johansen, Tarje Onsøien Halvorsen, Hong Yan Dai, Sissel Gyrid Freim Wahl, Elisabeth Fritzke Emdal, Bjørn Henning Grønberg

**Affiliations:** 1 Department of Clinical and Molecular Medicine, Faculty of Medicine and Health Sciences, Norwegian University of Science and Technology (NTNU), Trondheim, 7030 Norway; 2 Department of Oncology, St. Olavs Hospital, Trondheim University Hospital, Trondheim, 7030 Norway; 3 Department of Radiology and Nuclear Medicine, St. Olavs Hospital, Trondheim University Hospital, Trondheim, 7030 Norway; 4 Department of Pathology, Clinic of Laboratory Medicine, St. Olavs Hospital, Trondheim University Hospital, Trondheim, 7030 Norway

**Keywords:** ^18^F-FDG PET/CT, Circulating tumor DNA, Non-small cell lung cancer, Glucose metabolism, Liquid biopsy

## Abstract

**Background:**

The low level of circulating tumor DNA (ctDNA) in the blood is a well-known challenge for the application of liquid biopsies in early-stage non-small cell lung cancer (NSCLC) management. Studies of metastatic NSCLC indicate that ctDNA levels are associated with tumor metabolic activity as measured by ^18^F-fluorodeoxyglucose positron emission tomography (^18^F-FDG PET/CT). This study investigated this association in NSCLC patients considered for potentially curative treatment and explored whether the two methods provide independent prognostic information.

**Method:**

Patients with stage I-III NSCLC who had routinely undergone an ^18^F-FDG PET/CT scan and exploratory ctDNA analyses were included. Tumor glucose uptake was measured by maximum standardized uptake value (SUVmax), metabolic tumor volume (MTV), and total lesion glycolysis (TLG) from the ^18^F-FDG PET/CT scans. ctDNA detectability and quantity, using variant allele frequency, were estimated by tumor-informed ctDNA analyses.

**Results:**

In total, 63 patients (median age 70 years, 60% women, and 90% adenocarcinoma) were included. The tumor glucose uptake (SUVmax, MTV, and TLG) was significantly higher in patients with detectable ctDNA (n = 19, p < 0.001). The ctDNA quantity correlated with MTV (Spearman’s ρ = 0.53, p = 0.021) and TLG (Spearman’s ρ = 0.56, p = 0.013) but not with SUVmax (Spearman’s ρ = 0.034, p = 0.15). ctDNA detection was associated with shorter OS independent of MTV (HR: 2.70, 95% CI: 1.07–6.82, p = 0.035) and TLG (HR: 2.63, 95% CI: 1.06–6.51, p = 0.036). Patients with high tumor glucose uptake and detectable ctDNA had shorter overall survival and progression-free survival than those without detectable ctDNA, though these associations were not statistically significant (p > 0.05).

**Conclusion:**

There was a positive correlation between plasma ctDNA quantity and MTV and TLG in early-stage NSCLC patients. Despite the correlation, the results indicated that ctDNA detection was a negative prognostic factor independent of MTV and TLG.

**Supplementary Information:**

The online version contains supplementary material available at 10.1186/s12885-023-11147-z.

## Introduction

The analysis of tumor-specific mutations in circulating tumor DNA (ctDNA) can provide diagnostic and prognostic information in non-small cell lung cancer (NSCLC) [1]. The low ctDNA quantity in plasma is, however, a well-known limitation of the utility of ctDNA analysis in NSCLC patients receiving potentially curative treatment [2].

Previous studies, including studies on NSCLC, have reported that high ctDNA quantity is associated with high tumor metabolic activity, which can be estimated by ^18^F-fluorodeoxyglucose positron emission tomography (^18^F-FDG PET/CT) [1, 3–12]. By this method, the glucose uptake level in the tumor region is semi-quantified as the standardized uptake value (SUV), and used to identify malign lesions based on their higher-than-normal SUV. ^18^F-FDG PET/CT is routinely used to accurately assess the extent of disease in NSCLC patients eligible for potentially curative therapy, though the high normal glucose uptake in the brain limits the ability to detect brain metastases. In addition, the metabolic tumor volume (MTV) and total lesion glycolysis (TLG) can also be derived from the ^18^F-FDG PET/CT scans. The highest SUV in the lesion (SUVmax), MTV, and TLG are candidate prognostic factors in NSCLC [13].

The association between the glucose uptake level and ctDNA quantity is interesting for two reasons. First, it may contribute to a better understanding of what characterizes patients with detectable ctDNA and thus, identify those who might benefit from ctDNA analyses. Second, ctDNA analyses and ^18^F-FDG PET/CT-derived parameters might provide overlapping prognostic information. This is especially relevant for early-stage NSCLC for which ^18^F-FDG PET/CT is routinely performed, potentially limiting the prognostic value of ctDNA analyses. On the other hand, ctDNA might support findings on ^18^F-FDG PET/CT scans and aid the interpretation, especially when lesions with low ^18^F-FDG uptake are seen. Few studies have investigated the association between ctDNA detection and glucose uptake in early-stage NSCLC.

This study explored associations between tumor glucose uptake (measured by SUVmax, MTV, and TLG) and both ctDNA detectability and quantity in patients considered for potentially curative treatment. Furthermore, we explored whether ctDNA detection and ^18^F-FDG PET/CT-derived parameters were independent prognostic factors.

## Methods

### Study population, approvals, and data collection

Biological material was retrieved from the regional research biobank, Biobank1, approved by the Norwegian Regional Committee for Medical and Health Research Ethics in Central Norway, the Ministry of Health and Care Services, and the Norwegian Data Protection Authority. Biobank participants were 18 years or older and gave written informed consent. Patients were treated and followed according to local routines.

The present study included patients with stage I-III NSCLC from three previous studies on ctDNA who had available ^18^F-FDG PET/CT scans obtained during their diagnostic workup. Clinical data were collected from the patient’s hospital medical records, which included accurate survival data. The disease stage was assessed according to TNM v8 [14].

### ctDNA-analyses

ctDNA data was available from three previous studies [15–17]. In one cohort, tumor tissue DNA was screened for a pathogenic mutation in the gene *Kirsten Rat Sarcoma Viral Oncogene Homolog* (*KRAS*) [18]. Tumor tissue DNA in the other cohorts were screened for pathogenic mutations in 22[17] or 275[16] genes using next-generation sequencing (NGS). Tumor-informed analyses of ctDNA were performed in these studies using digital droplet polymerase chain reaction (ddPCR)[18] or NGS,[16, 17] and detection was defined as identifying ≥ one tumor-specific mutation(s) in ctDNA. ctDNA was quantified using the variant allele frequency.

### ^18^F-FDG PET/CT scans

^18^F-FDG PET/CT was not available at our hospital until autumn 2013. Thus, patients underwent ^18^F-FDG PET/CT at three different hospitals: Haukeland University Hospital, Bergen (n = 3), Oslo University Hospital, Oslo (n = 9), and St. Olav’s Hospital, Trondheim (n = 51). All hospitals used scanners from Siemens Healthcare (Erlangen, Germany), specifically the Biograph 40 in Bergen, the Biograph 64 in Oslo and the Biograph mCT 64 in Trondheim. The European Association of Nuclear Medicine (EANM) granted an EANM Research Ltd. (EARL) ^18^F-FDG PET/CT accreditation in September 2015 for the ^18^FDG PET/CT scanner at St. Olavs University Hospital. The EARL accreditation status for the other centers between 2011 and 13 is unknown.

Image reconstruction was performed with iterative reconstruction, point-spread-function (PSF), decay-, attenuation-, and scatter-correction. Time-of-flight (TOF) was used when available. Different matrix sizes were applied at different sites. All examinations were done following the EANM procedure guidelines for tumor imaging version 2.0 [19]. Patients fasted at least four hours (median 14 h) before administration of 4 MBq ^18^F-FDG/kg. Blood glucose level was 4.4–9.5 mmol/L (median 5.6 mmol/L), and the interval between ^18^F-FDG administration and the start of the acquisition was 51–159 min (median 60 min). A low-dose CT for attenuation correction and anatomical localization was done in the same session.

Datasets were transferred from the hospital’s picture archiving and communication systems and reprocessed using standard clinical software (AW Server 3.2 Ext. 3.0, General Electric Company) by a nuclear medicine physician (HJ). The physician was blinded for the ctDNA data but not the previous ^18^F-FDG PET/CT reports. A 3D isocontour model with a threshold of SUV of 2.5 was used when computing MTV and TLG (= product of MTV and SUVmean). MTV and TLG were calculated manually in separate sessions for each lesion when ^18^F-FDG uptake from lesions conflated. The highest value of SUVmax in any lesion was used for each patient. For both MTV and TLG, the sum of all lesions was used for statistical analyses. The raw PET data were not available and thus, the original AC-PET reconstructions were used to assess MTV and TLG.

### Statistics

SUVmax was compared between patients with and without detectable ctDNA using the Mann-Whitney U test since the values were not normally distributed. Spearmans’ correlation was used to investigate the correlation between SUVmax and the ctDNA quantity, measured by the highest variant allele frequency in cases of > 1 variant. Logistic multivariable regression models included SUVmax (continuous), histology, and disease stage to investigate the association between tumor glucose uptake and ctDNA detection. All analyses were repeated for MTV and TLG.

PFS was defined as the time from lung cancer diagnosis until progression or death of any cause, and OS was defined as the time from diagnosis until death of any cause. The median follow-up times for PFS and OS were estimated using the reverse Kaplan-Meier method, and the median PFS and OS were estimated using the Kaplan-Meier method. The impacts of ctDNA detectability and SUVmax on OS and PFS were estimated by univariable Cox proportional hazard models. To investigate the relationship between only ctDNA analysis and ^18^F-FDG PET/CT as prognostic factors, multivariable models for PFS and OS were performed, including SUVmax as a continuous factor and ctDNA detection. Additionally, Cox proportional hazard analyses were performed for PFS and OS including age, sex, WHO performance status, disease stage, histology, treatment modality, ctDNA detection and SUVmax. The combined prognostic value of ctDNA detection and tumor glucose uptake was explored in patients with high SUVmax (> median value in our cohort) by comparing outcomes between those with and without detectable ctDNA using the Kaplan-Meier method. All analyses of OS and PFS were repeated for MTV and TLG.

Statistical analyses were performed using R (version 3.6.1) with 0.05 as the threshold for statistical significance.

## Results

### Patient characteristics

In total, 63 patients diagnosed between July 2009 and May 2018 met the eligibility criteria for the present study (Table 1). The median age was 70, 38 (60%) were female, and 59 (94%) were smokers or former smokers. Fifty-seven patients (90%) had adenocarcinoma, two (3%) had squamous cell carcinoma, three (5%) had NSCLC not otherwise specified, and one (2%) had large cell neuroendocrine carcinoma. Twenty-eight patients (44%) had stage I disease, 12 (19%) stage II, and 23 (37%) stage III. ctDNA was detected in plasma from 19 patients (30%). Patients with detectable ctDNA had higher disease stage and median MTV, TLG, SUVmax, and lower surgical rate than those without detectable ctDNA. Otherwise, patient characteristics were similar between the two groups.

**Table 1:** Patient characteristics

	All patients		ctDNA detected		ctDNA not detected	
Total	63		19		44	
Age (median)	70	(52–83)	68	(52–83)	70	(52–81)
**Sex**						
Female	38	60%	11	58%	27	61%
Male	25	40%	8	42%	17	39%
**Smoking status**						
Smoker/former smoker	59	94%	17	89%	42	95%
Never smoker	4	6%	2	11%	2	5%
**Histology**						
Adenocarcinoma	57	90%	16	84%	41	93%
Non-adenocarcinoma	6	10%	3	16%	3	7%
**WHO performance status**						
0	37	59%	11	58%	26	59%
1	23	37%	8	42%	15	34%
2	2	3%	0	0%	2	5%
3	1	2%	0	0%	1	2%
**Disease stage**						
I	28	44%	0	0%	28	64%
II	12	19%	5	26%	7	16%
III	23	37%	14	74%	9	20%
**Treatment**						
Surgery	48	76%	8	42%	40	91%
Curative radiotherapy with or without chemotherapy	11	17%	9	47%	2	5%
Palliative therapy	4	6%	2	11%	2	5%
**ctDNA detection method**						
NGS^*^	33	52%	13	68%	20	45%
ddPCR	30	48%	6	32%	24	55%
^**18**^ ** F-FDG PET/CT parameters (median)**						
MTV (cm^3^)	7.5		61.2		3.7	
TLG (g/mL x cm^3^)	39.1		460.5		14.8	
SUVmax (g/mL)	11.8		19.2		9.1	

*One patient was included from a study analyzing ctDNA by a 275 NGS gene panel, and cfDNA from the other 32 patients was analyzed by patient-specific NGS panels. ddPCR: droplet digital polymerase chain reaction, NGS: next-generation sequencing, MTV: metabolic tumor volume, SUV: standardized uptake value, TLG: total lesion glycolysis

### Tumor glucose uptake in patients with and without detectable ctDNA

**Fig. 1:**
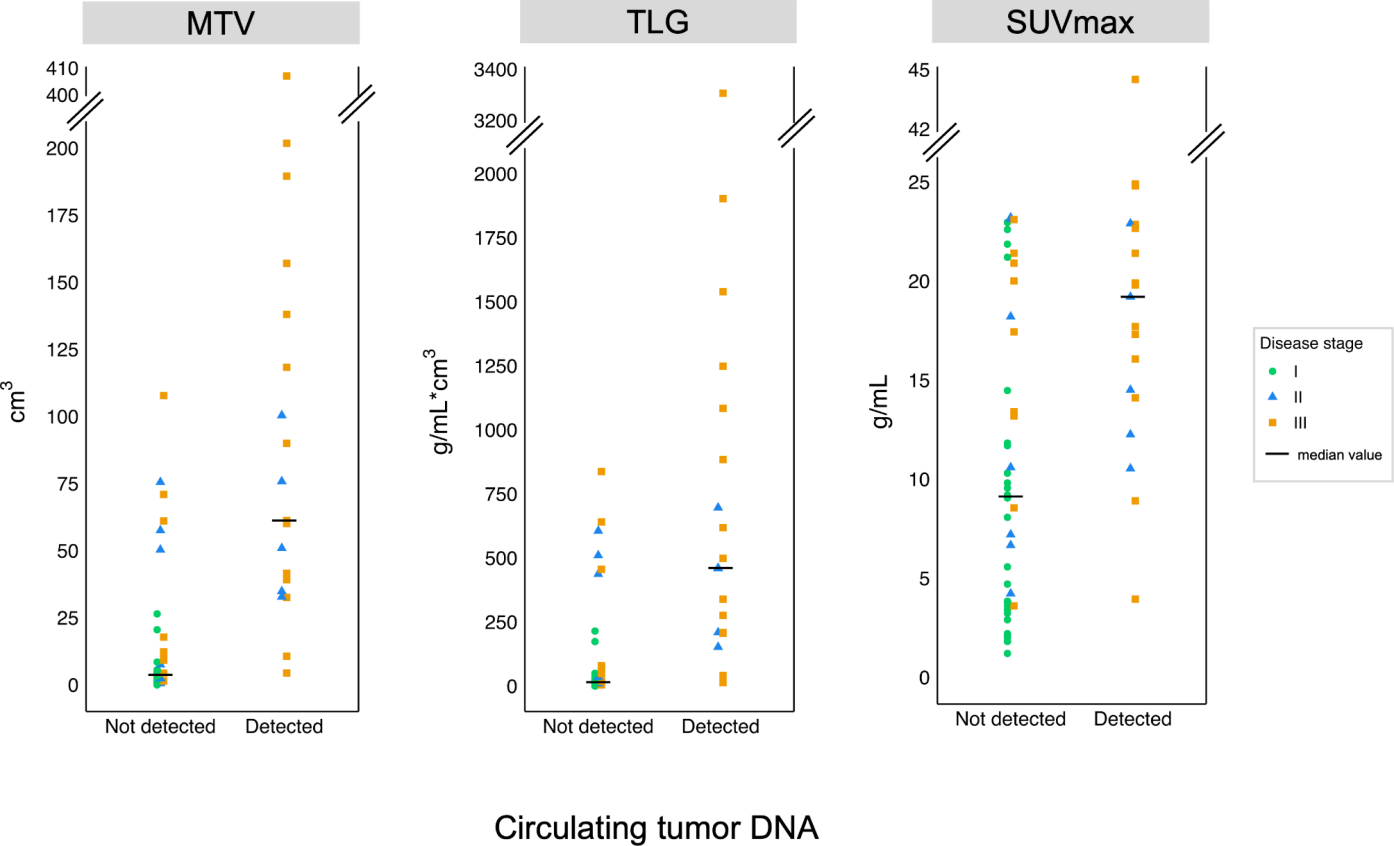
MTV, TLG and SUVmax derived from ^18^F-FDG PET/CT scans from patients with and without detectable ctDNA. ctDNA: circulating tumor DNA, MTV: metabolic tumor volume, TLG: total lesion glycolysis, SUVmax: maximum standardized uptake value

Patients with detectable ctDNA had significantly higher MTV (p < 0.001), TLG (p < 0.001), and SUVmax (p < 0.001) than patients without detectable ctDNA (Fig. 1).

**Fig. 2:**
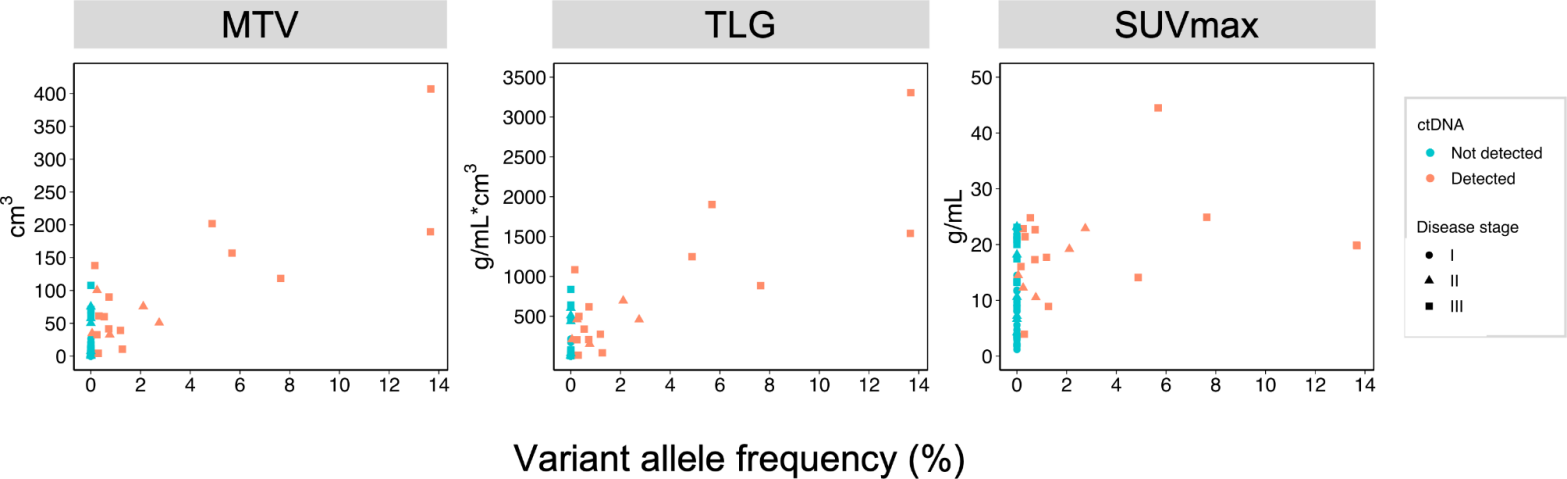
MTV, TLG, SUVmax and the ctDNA quantity, measured as the highest variant allele frequency

The ctDNA quantity correlated with MTV (Spearman’s ρ = 0.53, p = 0.0211) and TLG (Spearman’s ρ = 0.56, p = 0.0127), but not with SUVmax (Spearman’s ρ = 0.34, p = 0.15) in patients with detectable ctDNA (Fig. 2).

.

### Tumor glucose uptake as a predictor of ctDNA detection

Higher MTV (OR 19.3, 95% CI: 5.4-116.9, p < 0.001), TLG (OR 9.0, 95% CI: 3.4–33.8, p < 0.001), and SUVmax (OR 47.3, 95% CI: 5.2-937.4, p = 0.0030) were associated with ctDNA detection in univariable logistic regression analyses. MTV (OR 1.03, 95% CI: 1.01–1.05, p = 0.019) remained associated with ctDNA detection independent of disease stage and histology in multivariable analysis (Table 2). Similarly, TLG (OR 1.00, 95% CI: 1.00-1.01, p = 0.038) was independently associated with ctDNA detection, while SUVmax was not (OR: 1.07, 95% CI: 0.98–1.20, p = 0.19). Since ctDNA was not detected in stage I patients, sensitivity analyses including only stage II-III patients were performed, with similar results (Table S1).

**Table 2:** Multivariable logistic regression models with ctDNA detection as a response

	MTV			TLG			SUVmax		
	OR	95% CI	*p*	OR	95% CI	*p*	OR	95% CI	*p*
Stage I	1.00			1.00			1.00		
Stage II	8.75E + 07	0-NA	0.99	1.14E + 08	0-NA	0.99	1.75E + 08	0-NA	0.99
Stage III	1.32E + 08	0-NA	0.99	1.76E + 08	0-NA	0.99	3.00E + 08	0-NA	0.99
Adenocarcinoma	1.00			1.00			1.00		
Non-adenocarcinoma	3.59	0.29–87.72	0.34	3.74	0.3-90.89	0.32	2.72	0.28–61.19	0.42
MTV	1.03	1.01–1.05	**0.019**	-	-	-	-	-	-
TLG	-	-	-	1.00	1.00-1.01	**0.038**	-	-	-
SUVmax	-	-	-	-	-	-	1.07	0.98–1.20	0.19

Statistically significant p-values are shown in bold. CI: confidence interval; ctDNA: circulating tumor DNA; MTV: metabolic tumor volume; OR: odds ratio, SUVmax: maximum standardized uptake value; TLG: total lesion glycolysis

### Tumor glucose uptake and ctDNA detection as prognostic factors

The median follow-up time for OS was 57.0 months (95% CI: 50.7–64.0), and 33 patients were alive at the time of analysis. Overall, median OS was not reached (95% CI: 39.9 months - not reached [NR]). Higher MTV (HR 1.00, 95% CI: 0.995-1.00, p = 0.017), TLG (HR: 1.00, 95% CI: 0.995-1.00, p = 0.017), and SUVmax (HR: 1.05, 95% CI: 0.948–1.02, p = 0.004) were associated with worse OS in univariable analyses (Table 3).

**Table 3:** Univariable Cox proportional hazard analyses of OS

	HR	95% CI	*p*
ctDNA detected	3.13	1.46–6.73	**0.0034**
MTV	1.00	1.00-1.01	**0.017**
TLG	1.00	1.00–1.00	**0.013**
SUVmax	1.05	1.02–1.09	**0.0036**

Statistically significant p-values are shown in bold. CI: confidence interval; ctDNA: circulating tumor DNA; HR: hazard ratio, MTV: metabolic tumor volume, SUVmax: maximum standardized uptake value; TLG: total lesion glycolysis

Multivariable analyses (Table 4) showed that ctDNA detection was associated with worse OS independently of MTV (HR: 2.70, 95% CI: 1.07–6.82, p = 0.035) and TLG (HR: 2.63, 95% CI: 1.06–6.51, p = 0.036), but not SUVmax (HR: 2.30, 95% CI: 0.977–5.42, p = 0.056). The ^18^F-FDG PET/CT-derived parameters were not independently associated with OS in the same models (MTV, HR: 1.00, 95% CI: 0.996–1.01, p = 0.55. TLG, HR: 1.00, 95% CI: 1.00–1.00, p = 0.43. SUVmax, HR: 1.03, 95% CI: 0.995–1.08, p = 0.087.) In multivariable analyses including established prognostic factors for NSCLC, neither ctDNA detection nor any ^18^F-FDG PET/CT-derived parameter was significantly associated with OS (Table S3).

**Table 4:** Multivariable Cox proportional hazard analyses of OS

	MTV			TLG			SUVmax		
	HR	95% CI	*p*	HR	95% CI	*p*	HR	95% CI	*p*
ctDNA not detected	1.00			1.00			1.00		
ctDNA detected	2.70	1.07–6.82	**0.035**	2.63	1.06–6.51	**0.036**	2.30	0.977–5.42	0.056
MTV	1.00	0.996–1.01	0.55						
TLG				1.00	1.00–1.00	0.43			
SUVmax							1.03	0.995–1.08	0.087

Statistically significant p-values are shown in bold. CI: confidence interval; ctDNA: circulating tumor DNA; HR: hazard ratio, MTV: metabolic tumor volume, SUVmax: maximum standardized uptake value; TLG: total lesion glycolysis

The median follow-up time for PFS was 57.0 months (95% CI: 50.7–65.6), and 29 patients were alive and progression-free at the time of analysis. The median PFS was 61.8 months (95% CI: 19.1-NR). ctDNA detection and higher MTV, TLG, and SUVmax were significantly associated with worse PFS in univariable Cox proportional hazard analyses (p < 0.05, Table S2). None of the factors were independently associated with PFS in multivariable analyses (Table S4 and S5).

### Combined prognostic value of ctDNA analyses and ^18^F-FDG PET/CT-derived parameters

**Fig. 3:**
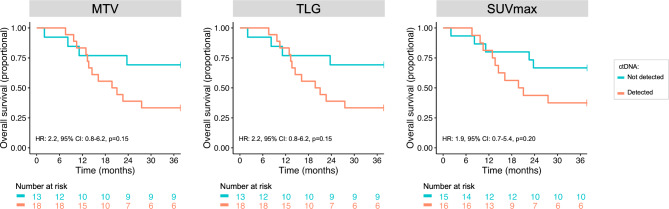
Kaplan-Meier plots showing OS for patients with A: MTV, B: TLG and C: SUVmax above the median value and split on ctDNA status

Among patients with MTV above median value, those with detectable ctDNA had shorter OS than those without detectable ctDNA (median 20.4 months vs. NR, Fig. 3), though the difference was not statistically significant (HR: 2.2, 95% CI: 0.8–6.2, p = 0.15). A similar difference was observed among patients with TLG above median value (median 20.4 months vs. NR, HR: 2.2, 95% CI: 0.8–6.2, p = 0.15) and SUVmax above median value (median 20.4 months vs. NR, HR: 1.9, 95% CI: 0.7–5.4, p = 0.202). Similarly, among patients with MTV, TLG, or SUVmax above the median value, those with detectable ctDNA had shorter PFS than those without detectable ctDNA, though the differences were not statistically significant (Figure S1). There were too few patients (n < 4) with detectable ctDNA among those with MTV, TLG, or SUVmax below median values to perform such analyses.

## Discussion

In this study of patients with stage I-III NSCLC considered for potentially curative therapy, we found that tumor glucose uptake was significantly higher in patients with detectable ctDNA, and the ctDNA quantity correlated with MTV and TLG. Nevertheless, ctDNA detection was a negative prognostic factor for OS independently of the ^18^F-FDG PET/CT-derived parameters.

There is limited research on the association between ^18^F-FDG PET/CT and ctDNA detection in early-stage NSCLC. Our results reflect those of Chabon et al., who included 85 early-stage NSCLC patients and found that those with detectable ctDNA had higher MTV and that ctDNA quantity correlated with MTV [12]. In addition, they found that ctDNA detection was a negative prognostic factor independent of both MTV and disease stage. Another study of 92 patients enrolled in the TRACERx study found that high ^18^F-FDG avidity, defined as the ratio between tumor and mediastinal SUVmax, predicted ctDNA detection [11]. That study did not investigate whether the prediction was independent of other patient and disease characteristics. While SUVmax was significantly higher in patients with detectable ctDNA in our study, it was not independently associated with ctDNA detection after adjusting for disease stage and histology.

Several studies have investigated the association between ctDNA characteristics and all three ^18^F-FDG PET/CT-derived parameters in advanced NSCLC [1, 4, 6, 7]. Although these are mostly small studies (n = 37–128) with methodological variations, they indicate a positive correlation between ctDNA release and glucose uptake. In agreement with our results, one study reported a correlation between ctDNA quantity and MTV and TLG but not with SUVmax [4]. Other studies found a positive correlation with SUVmax as well,[6, 7] such as the recent study of Jee and colleagues which included 128 advanced NSCLC patients, the largest study to date [1].

A few studies on advanced NSCLC found no correlation between ctDNA release and tumor metabolic activity [7, 8, 20]. Common for these studies is that they analyzed the total cfDNA quantity rather than the mutant ctDNA fraction. Notably, González de Aledo-Castillo et al. observed that cfDNA at 100–250 bp length, which includes the typical length of ctDNA, correlated with glucose uptake, while total cfDNA quantity did not [8]. The total cfDNA quantity may be influenced by non-cancer related factors [21].

We and others observed cases of undetectable or low ctDNA quantity but high glucose uptake and vice versa [1, 3, 5, 6, 8, 12]. Although the two variables usually correlate, evidence from studies on NSCLC suggests that ctDNA analysis and ^18^F-FDG PET/CT may provide independent prognostic information [1, 3, 5, 12]. Studies of metastatic cancers, including NSCLC, have also indicated a combined value of the two analyses [1, 3, 9, 10]. For example, Jee et al. demonstrated that ctDNA detection was a negative prognostic factor both for patients with high and low glucose uptake [1]. The high number of stage I patients without detectable ctDNA was the reason for not investigating the prognostic role of detectable ctDNA among our patients with low glucose uptake levels.

The results of this study must be interpreted in the context of its limitations and strengths. This was a retrospective study of mostly adenocarcinoma patients, of which 56% had a *KRAS* mutation, compared to ~ 38% in the Norwegian lung adenocarcinoma population [18]. The sample size did not allow adjusting for other established prognostic factors such as disease stage, performance status, or therapy in the multivariable OS and PFS analyses. Additionally, tumor characteristics associated with ctDNA detectability, such as proliferation rate, the extent of necrosis, and vascular infiltration, were not assessed.

The ^18^F-FDG PET/CT scans were performed on several scanners and sites using various dosages of ^18^F-FDG/kg, though 80% of scans were performed on the scanner at our site using the same protocol. The values of the ^18^F-FDG PET/CT-derived parameters are, in principle, dependent on the characteristics of the PET/CT camera, reconstruction parameters, matrix size, and PSF, which could cause a batch effect. Although raw data was not available from the other hospitals and different parameters may have influenced the calculated values, we do not believe that a potential batch effect has significantly influenced the overall result. The variation in MTV and TLG was less than 5% when values assessed locally were compared with centrally reconstructed parameters, and measures were taken to compensate for the partial volume effects, including the tissue fraction effects. Importantly, no tumors in our study were < 3 mm, limiting the risk of SUVmax underestimation. It is unclear which ^18^F-FDG PET/CT variable holds the most prognostic information, but most other studies have used the same variables as we did [1, 3, 4, 6, 7]. Finally, we did not have information about co-existing conditions (e.g. sarcoidosis) or medications that might have influenced the ^18^F-FDG uptake.

The lack of standardized methods for ctDNA detection and accurate quantification is a general challenge for ctDNA research. Using the variant allele frequency for ctDNA quantification is common but not optimal since the frequency depends on the total cfDNA quantity.

The high sensitivity and specificity are strengths of the tumor-informed approach applied for ctDNA analysis in this study. Nevertheless, we cannot rule out the possibility of false negatives. Another study detected ctDNA in 45% of early-stage NSCLC patients and noted that the likelihood of ctDNA detection increased with the number of analyzed mutations [12]. The tumor DNA screening limited the number of mutations for ctDNA analysis, and there was only knowledge of one mutation in the *KRAS*-positive cohort. Other contributing factors to the low detection rate may be the high proportion and stage I patients and adenocarcinomas, which probably release less ctDNA than other types of NSCLC [11].

The inclusion of early-stage patients was, in our opinion, the main feature of our study. Although many lower-stage NSCLC patients are cured by surgery and radiotherapy, the relapse rates are still relatively high. The effectiveness of adjuvant *EGFR* tyrosine kinase inhibitors and immunotherapy has recently been demonstrated, but similar to adjuvant chemotherapy, the absolute survival benefit is limited. There is an unmet need for tools identifying those with the highest risk of relapse who should be offered such adjuvant therapy to reduce the number of patients receiving unnecessary medication [22, 23]. One might argue that the clinical implications of the prognostic information of ctDNA detection and ^18^F-FDG PET/CT variables are fewer since most of these patients receive treatment anyway. In that setting, biomarkers predicting outcomes of specific treatments are more important.

The question remains what is the relationship between ctDNA release and tumor metabolism. It is important to remember that ^18^F-FDG PET/CT estimates the level of tumor glucose uptake and cannot be used to explain the metabolic state of the tumor. Elevated glucose uptake can reflect an elevated level of aerobic metabolism or a shift towards anaerobic metabolism due to hypoxia or the Warburg effect. Studies on NSCLC have indicated that squamous cell carcinomas are associated with anaerobic metabolism and adenocarcinomas with aerobic metabolism [24]. According to a recent publication, elevated glucose uptake might not reflect elevated metabolic activity at all [25]. The authors demonstrated that solid tumors in mice have a high glucose uptake without an increase in energy production and suggested that this is tolerated by the tumor cells by shutting down energy-costly tissue-specific processes. It is yet to be understood whether tumors in these different metabolic states have a similar rate of ctDNA release and whether the prognostic meaning of its release remains the same.

## Conclusion

We found a positive correlation between plasma ctDNA quantity and tumor glucose uptake measured by ^18^F-FDG PET/CT in early-stage NSCLC patients. Nevertheless, the result indicated that ctDNA analysis provided independent prognostic information from ^18^F-FDG PET/CT and larger studies are needed to investigate if there is a combined prognostic value of the two analyses. Furthermore, there is a need for a better understanding of the mechanism behind ctDNA release and the biological rationale behind the potential prognostic impact since it cannot be explained by the tumor glucose uptake alone.

## Electronic supplementary material

Below is the link to the electronic supplementary material.

Supplementary Material 1

Supplementary Material 2

## Data Availability

The Norwegian Health Research Act prevents us from sharing any clinical data, this includes information about the individual patient, the ^18^F-FDG PET/CT scans and ctDNA BAM files.

## List of abbreviations

ctDNA: circulating tumor DNA
^18^F-FDG PET/CT: ^18^F-fluorodeoxyglucose positron emission tomography
MTV: metabolic tumor volume
NSCLC: non-small cell lung cancer
OS: overall survival
PFS: progression-free survival
TLG: total lesion glycolysis
SUVmax: maximum standardized uptake value

## References

[1] Jee J, Lebow ES, Yeh R, Das JP, Namakydoust A, Paik PK (2022). Overall survival with circulating tumor DNA-guided therapy in advanced non-small-cell lung cancer. Nat Med.

[2] Abbosh C, Birkbak NJ, Swanton C (2018). Early stage NSCLC — challenges to implementing ctDNA-based screening and MRD detection. Nat Rev Clin Oncol.

[3] Hyun MH, Lee ES, Eo JS, Kim S, Kang EJ, Sung JS (2019). Clinical implications of circulating cell-free DNA quantification and metabolic tumor burden in advanced non-small cell lung cancer. Lung Cancer.

[4] Fiala O, Baxa J, Svaton M, Benesova L, Ptackova R, Halkova T (2022). Combination of circulating Tumour DNA and ^18^ F-FDG PET/CT for Precision Monitoring of Therapy response in patients with Advanced Non-small Cell Lung Cancer: a prospective study. Cancer Genomics - Proteomics.

[5] Winther-Larsen A, Demuth C, Fledelius J, Madsen AT, Hjorthaug K, Meldgaard P (2017). Correlation between circulating mutant DNA and metabolic tumour burden in advanced non-small cell lung cancer patients. Br J Cancer.

[6] Lam VK, Zhang J, Wu CC, Tran HT, Li L, Diao L (2021). Genotype-specific differences in circulating tumor DNA levels in Advanced NSCLC. J Thorac Oncol.

[7] Morbelli S, Alama A, Ferrarazzo G, Coco S, Genova C, Rijavec E (2017). Circulating Tumor DNA reflects Tumor Metabolism Rather Than Tumor Burden in Chemotherapy-Naive patients with Advanced non–small cell Lung Cancer: ^18^ F-FDG PET/CT study. J Nucl Med.

[8] González deAledo-Castillo, Jm, Casanueva-Eliceiry S, Soler-Perromat A, Fuster D, Pastor V, Reguart N (2021). Cell-free DNA concentration and fragment size fraction correlate with FDG PET/CT-derived parameters in NSCLC patients. Eur J Nucl Med Mol Imaging.

[9] Woff E, Kehagias P, Vandeputte C, Ameye L, Guiot T, Paesmans M (2019). Combining ^18^ F-FDG PET/CT–Based metabolically active tumor volume and circulating cell-free DNA significantly improves Outcome Prediction in Chemorefractory Metastatic Colorectal Cancer. J Nucl Med.

[10] Delfau-Larue M-H, van der Gucht A, Dupuis J, Jais J-P, Nel I, Beldi-Ferchiou A (2018). Total metabolic tumor volume, circulating tumor cells, cell-free DNA: distinct prognostic value in follicular lymphoma. Blood Adv.

[11] Abbosh C, Birkbak NJ, Wilson GA, Jamal-Hanjani M, Constantin T, Salari R (2017). Phylogenetic ctDNA analysis depicts early-stage lung cancer evolution. Nature.

[12] Chabon JJ, Hamilton EG, Kurtz DM, Esfahani MS, Moding EJ, Stehr H (2020). Integrating genomic features for non-invasive early lung cancer detection. Nature.

[13] Kaira K, Higuchi T, Naruse I, Arisaka Y, Tokue A, Altan B (2018). Metabolic activity by 18F–FDG-PET/CT is predictive of early response after nivolumab in previously treated NSCLC. Eur J Nucl Med Mol Imaging.

[14] Goldstraw P, Chansky K, Crowley J, Rami-Porta R, Asamura H, Eberhardt WEE (2016). The IASLC Lung Cancer Staging Project: proposals for revision of the TNM Stage Groupings in the Forthcoming (Eighth) Edition of the TNM classification for Lung Cancer. J Thorac Oncol.

[15] Wahl SGF, Dai HY, Emdal EF, Ottestad AL, Dale VG, Richardsen E (2021). Prognostic value of absolute quantification of mutated KRAS in circulating tumour DNA in lung adenocarcinoma patients prior to therapy. J Pathol Clin Res.

[16] Ottestad AL, Wahl SGF, Grønberg BH, Skorpen F, Dai HY. The relevance of tumor mutation profiling in interpretation of NGS data from cell-free DNA in non-small cell lung cancer patients. Exp Mol Pathol. 2019;:104347. 10.1016/j.yexmp.2019.104347 31759951

[17] Ottestad AL, Dai HY, Halvorsen TO, Emdal EF, Wahl SGF, Grønberg BH (2021). Associations between tumor mutations in cfDNA and survival in non-small cell lung cancer. Cancer Treat Res Commun.

[18] Wahl SGF, Dai HY, Emdal EF, Berg T, Halvorsen TO, Ottestad AL (2021). The Prognostic Effect of KRAS mutations in Non-Small Cell Lung Carcinoma Revisited: a norwegian Multicentre Study. Cancers.

[19] Boellaard R, Delgado-Bolton R, Oyen WJG, Giammarile F, Tatsch K, Eschner W (2015). FDG PET/CT: EANM procedure guidelines for tumour imaging: version 2.0. Eur J Nucl Med Mol Imaging.

[20] Nygaard AD, Holdgaard PC, Spindler K-LG, Pallisgaard N, Jakobsen A (2014). The correlation between cell-free DNA and tumour burden was estimated by PET/CT in patients with advanced NSCLC. Br J Cancer.

[21] Kananen L, Hurme M, Bürkle A, Moreno-Villanueva M, Bernhardt J, Debacq-Chainiaux F (2023). Circulating cell-free DNA in health and disease — the relationship to health behaviours, ageing phenotypes and metabolomics. GeroScience.

[22] Wu Y-L, Tsuboi M, He J, John T, Grohe C, Majem M (2020). Osimertinib in Resected *EGFR* -Mutated non–small-cell Lung Cancer. N Engl J Med.

[23] Felip E, Altorki N, Zhou C, Csőszi T, Vynnychenko I, Goloborodko O (2021). Adjuvant atezolizumab after adjuvant chemotherapy in resected stage IB–IIIA non-small-cell lung cancer (IMpower010): a randomised, multicentre, open-label, phase 3 trial. The Lancet.

[24] Schuurbiers OCJ, Meijer TWH, Kaanders JHAM, Looijen-Salamon MG, de Geus-Oei L-F, van der Drift MA (2014). Glucose metabolism in NSCLC is histology-specific and diverges the prognostic potential of 18FDG-PET for Adenocarcinoma and squamous cell carcinoma. J Thorac Oncol.

[25] Bartman CR, Weilandt DR, Shen Y, Lee WD, Han Y, TeSlaa T (2023). Slow TCA flux and ATP production in primary solid tumours but not metastases. Nature.

