# Correction: Associations between detectable circulating tumor DNA and tumor glucose uptake measured by ^18^F-FDG PET/CT in early-stage non-small cell lung cancer

**DOI:** 10.1186/s12885-023-11173-x

**Published:** 2023-07-20

**Authors:** Anine Larsen Ottestad, Håkon Johansen, Tarje Onsøien Halvorsen, Hong Yan Dai, Sissel Gyrid Freim Wahl, Elisabeth Fritzke Emdal, Bjørn Henning Grønberg

**Affiliations:** 1 Department of Clinical and Molecular Medicine, Faculty of Medicine and Health Sciences, Norwegian University of Science and Technology (NTNU), Trondheim, 7030 Norway; 2 Department of Oncology, St. Olavs Hospital, Trondheim University Hospital, Trondheim, 7030 Norway; 3 Department of Radiology and Nuclear Medicine, St. Olavs Hospital, Trondheim University Hospital, Trondheim, 7030 Norway; 4 Department of Pathology, Clinic of Laboratory Medicine, St. Olavs Hospital, Trondheim University Hospital, Trondheim, 7030 Norway

**Correction: BMC Cancer 23, 646 (2023)**

10.1186/s12885-023-11147-z

Following publication of the original article [1], the authors identified a typesetting error. Supplementary Material Fig. S2 was erroneously published as Fig. 2. The correct Fig. 2 is published in this correction article and the original article [1] has been corrected.

**Fig. 2:**
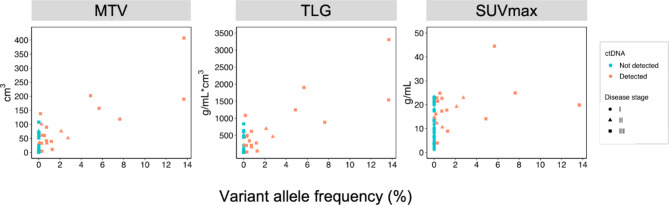
MTV, TLG, SUVmax and the ctDNA quantity, measured as the highest variant allele frequency
