# Supplementary material for: Associations between detectable circulating tumor DNA and tumor glucose uptake measured by 18F-FDG PET/CT in early-stage non-small cell lung cancer
Source: BMC Cancer. 2023 Jul 11;23:646. doi: 10.1186/s12885-023-11147-z (PMC10334612; doi:10.1186/s12885-023-11147-z)

Figure S1. Kaplan-Meier plots showing progression-free survival for patients with A: MTV, B: TLG and C: SUVmax above the median value and split on ctDNA status.

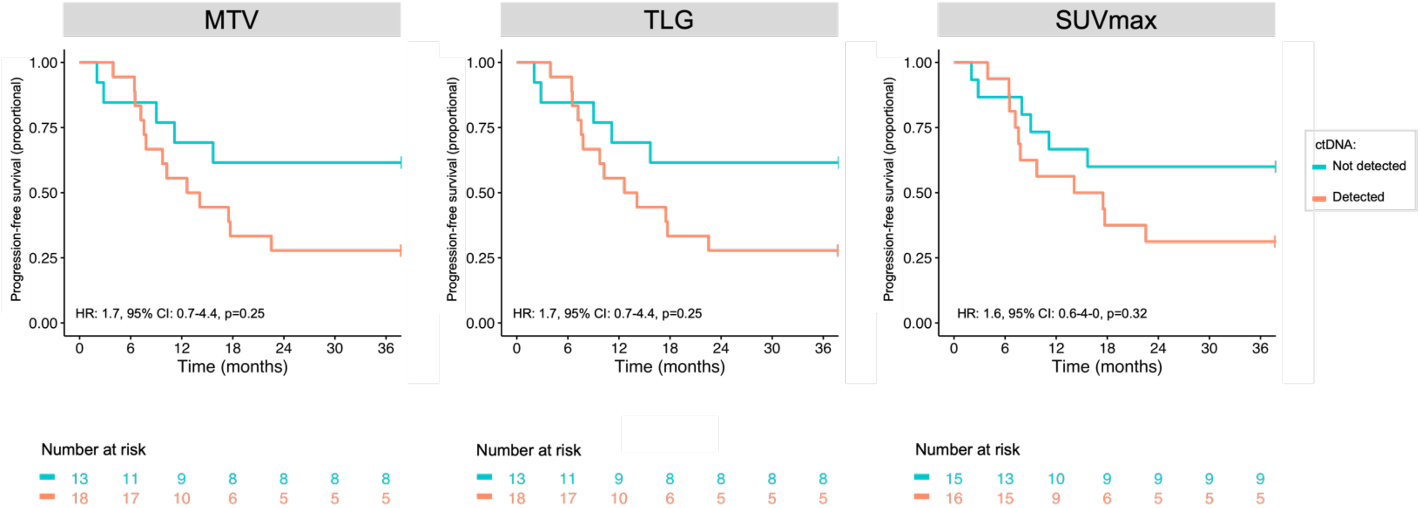

Supplement: Supplementary file 2 — Supplementary Material 2 [file 12885_2023_11147_MOESM2_ESM.pdf]
